# Supplementary material for: Isolation, Identification and Pharmacological Effects of Mandragora autumnalis Fruit Flavonoids Fraction
Source: Molecules. 2022 Feb 3;27(3):1046. doi: 10.3390/molecules27031046 (PMC8838059; doi:10.3390/molecules27031046)
Supplement: Supplementary file 1 [file molecules-27-01046-s001.zip › molecules-1550534-supplementary.pdf]

Supplementary Materials

Isolation, Identification and Pharmacological Effects of *Mandragora autumnalis* Fruit Flavonoids Fraction

Nawaf Al-Maharik [1,*], Nidal Jaradat [2,*], Najlaa Bassalat [3], Mohammed Hawash [2] and Hilal Zaid [4]

1 Division of Chemistry, Faculty of Science, An-Najah National University, Nablus 00970, Palestine
2 Department of Pharmacy, Faculty of Medicine and Health Sciences, An-Najah National University, Nablus 00970, Palestine; mohawash@najah.edu
3 Department of Biology and Biotechnology, Faculty of Sciences, Arab American University, Jenin 240, Palestine; nbassalat@gmail.com
4 Qasemi Research Center, Al-Qasemi Academic College, and Faculty of Medicine, Arab American University, Jenin 240, Palestine; hilal.zaid@gmail.com
* Correspondence: n.maharik@najah.edu (N.A.-M.); nidaljaradat@najah.edu (N.J.)

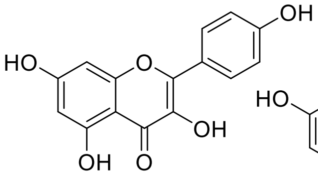

1

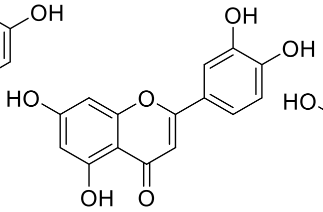

2

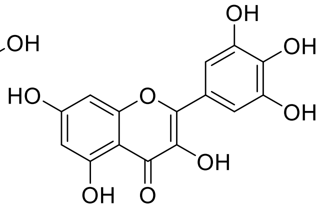

3

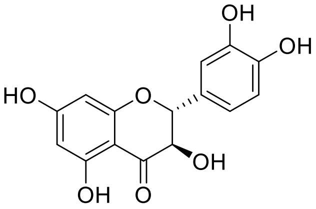

4

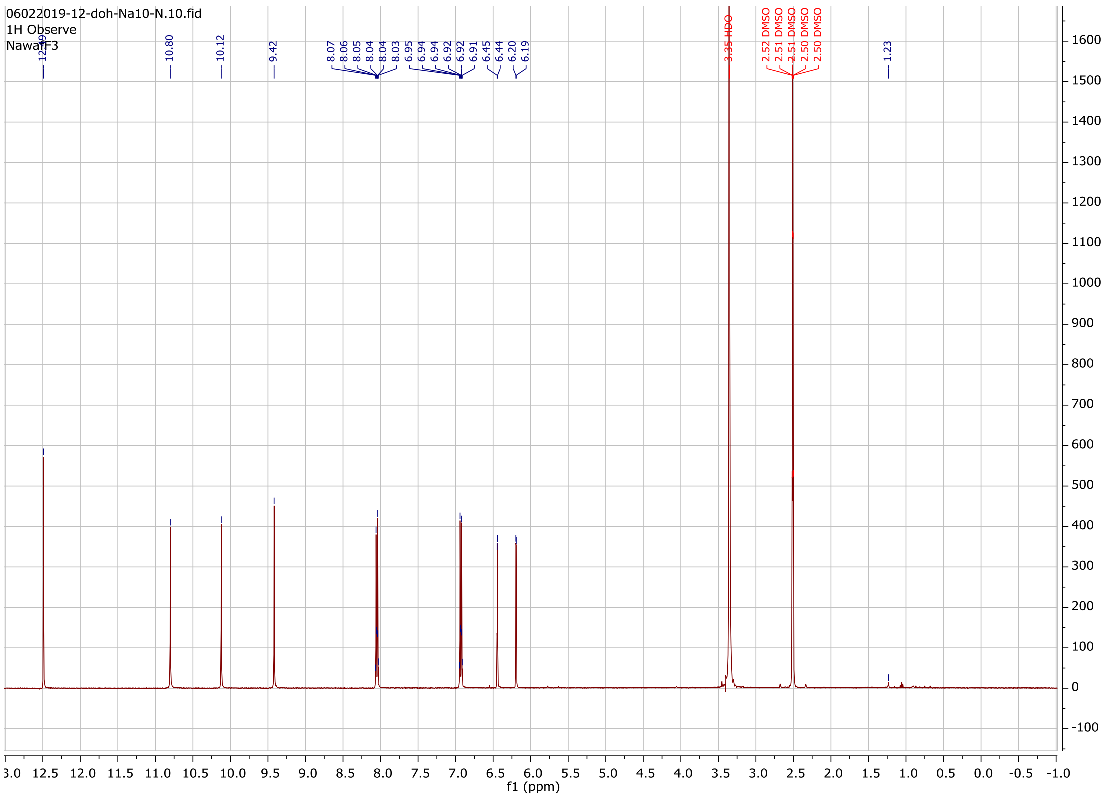

Figure S1. ^{1}H NMR spectrum of **1** in DMSO-d$_6$

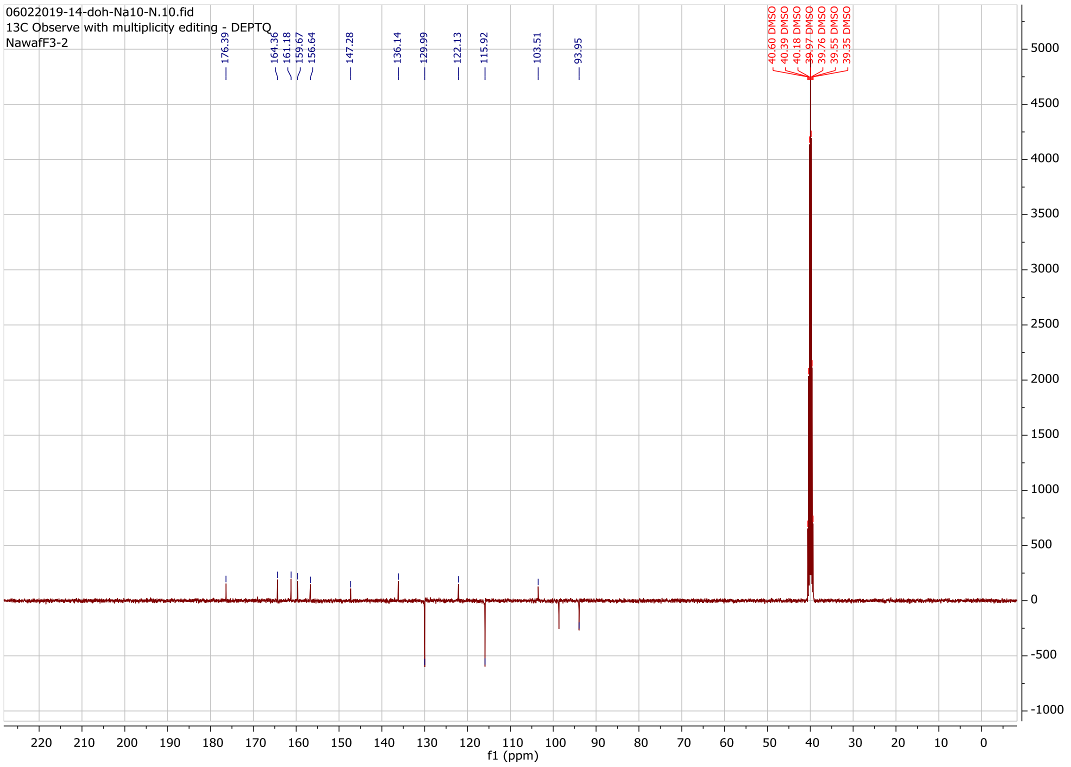

Figure S2. ^{13}C NMR spectrum (400 MHz) of **1** in DMSO-d$_6$

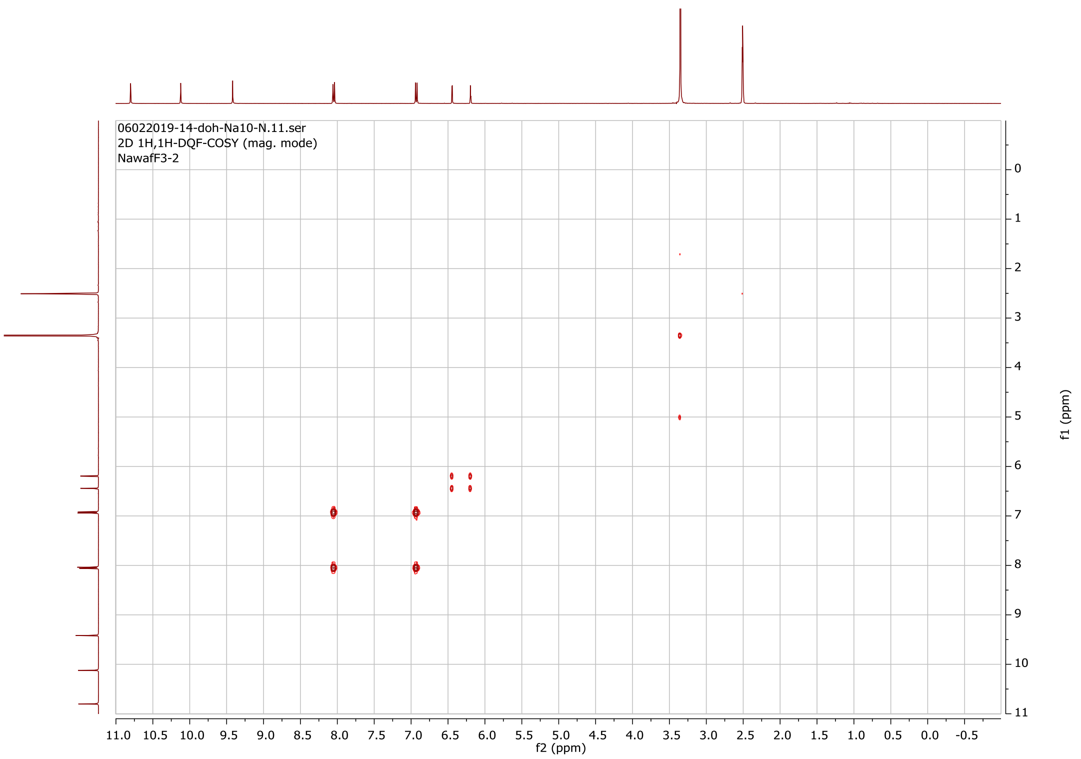

Figure S3. ¹H-¹H COSY spectrum of **1** in DMSO-d_6

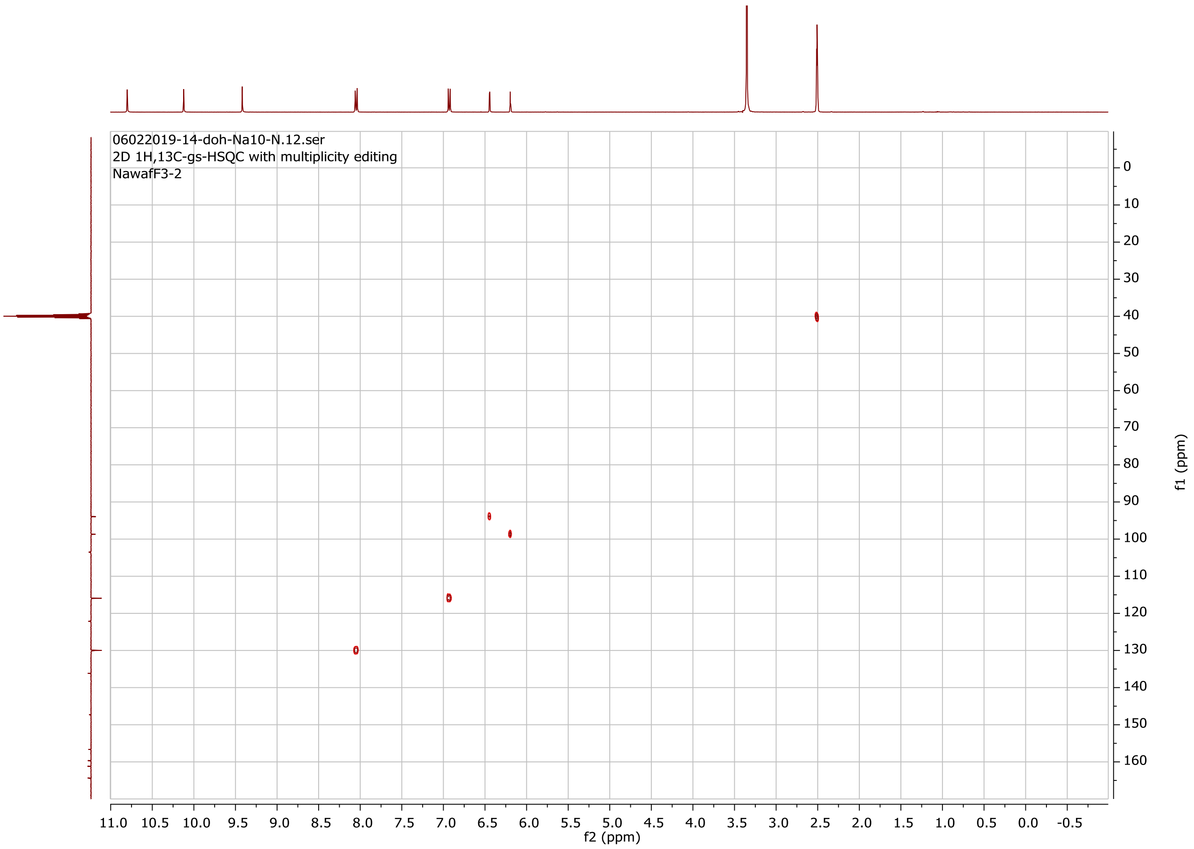

Figure S4. HSQC spectrum of **1** in DMSO-d_6.

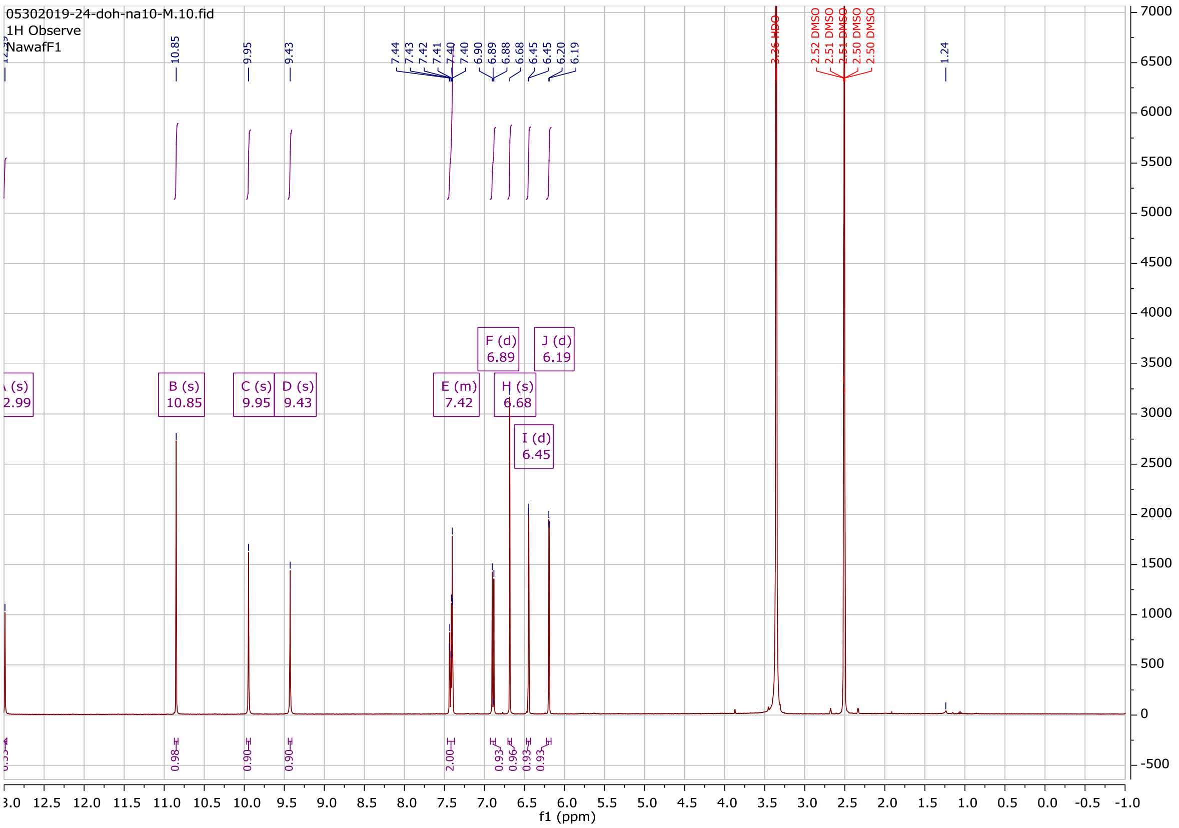

Figure S5. ^{1}H NMR spectrum of **2** in DMSO-d_6

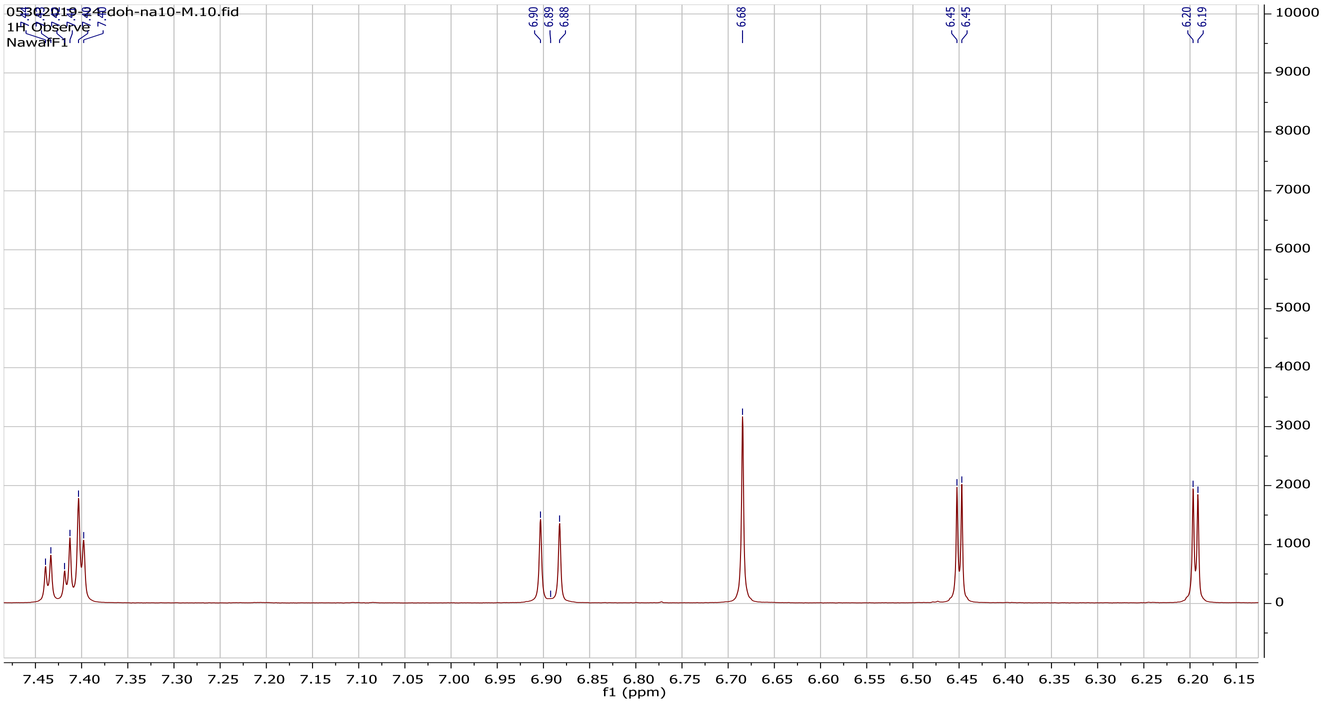

Figure S6. Expansion of the aromatic region of ^{1}H NMR spectrum of **2** in DMSO-d_6

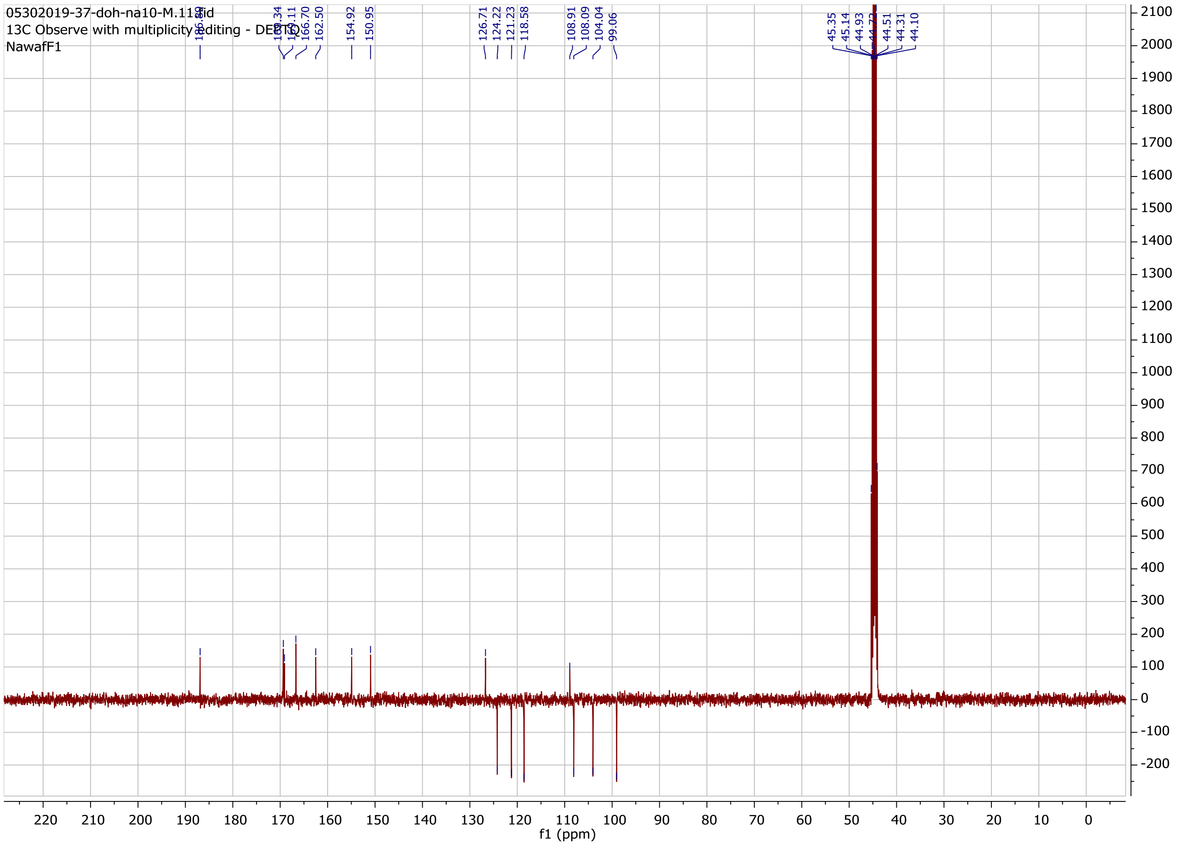

Figure S7. ^{13}C NMR (Dept) spectrum of **2** in DMSO-d$_6$

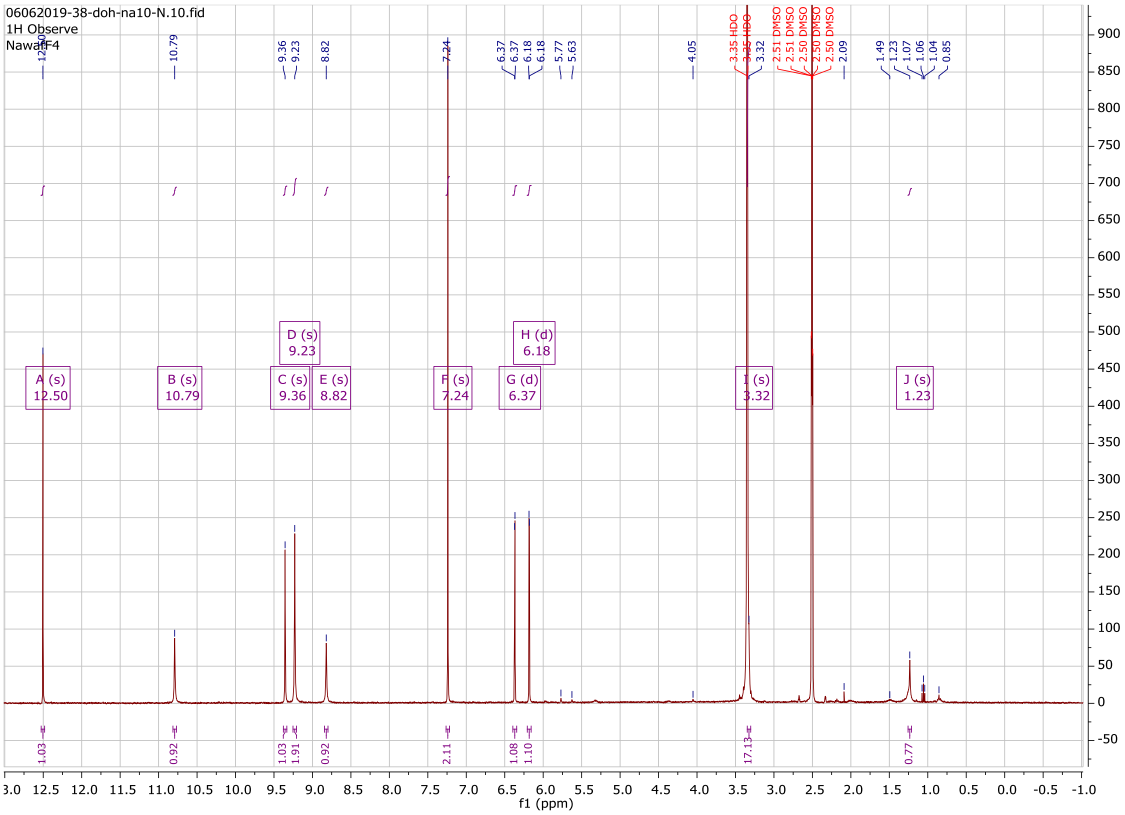

Figure S8. ^{1}H NMR spectrum of **3** in DMSO-d6

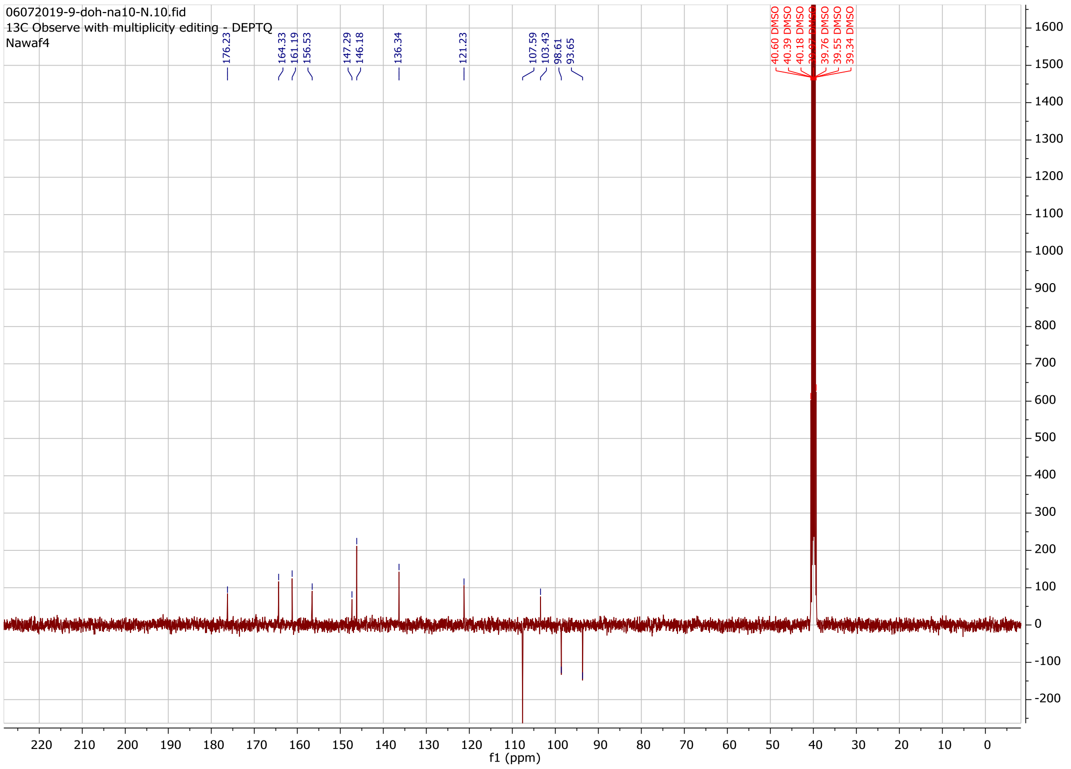

Figure S9. ^{13}C NMR (Dept) spectrum of **3** in DMSO-d6

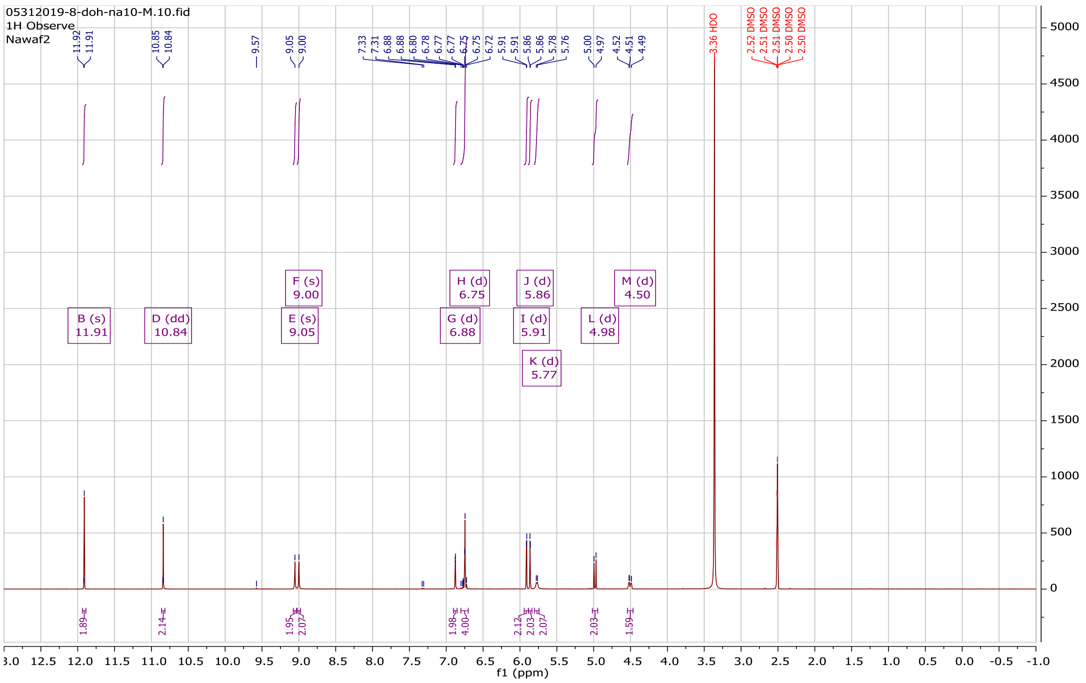

Figure S10. ^{1}H NMR spectrum of **4** in DMSO-d6

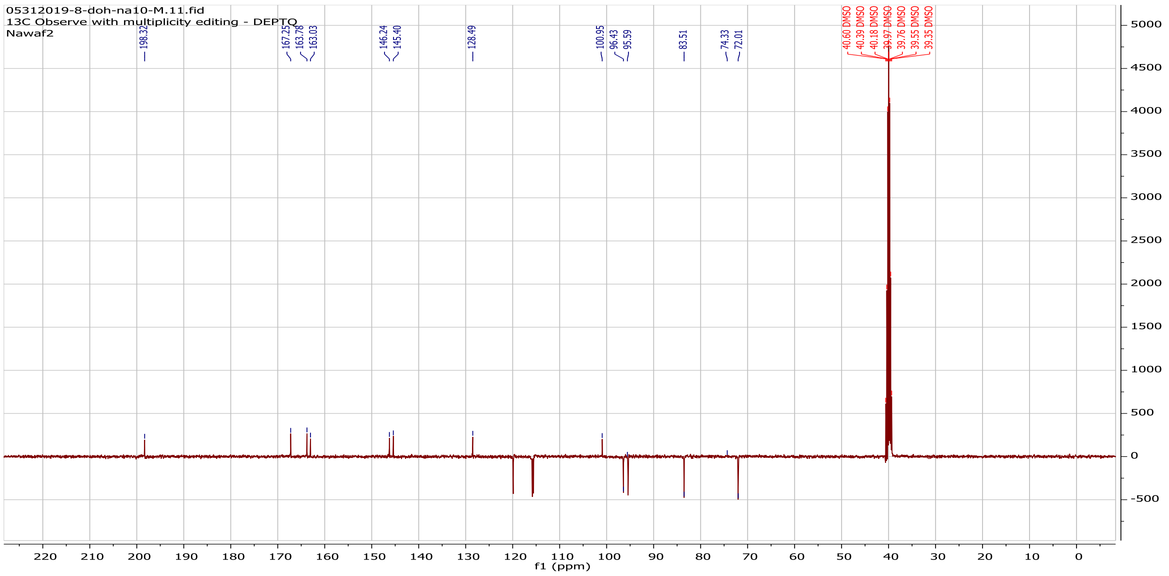

Figure S11. ^{13}C NMR (Dept) spectrum of **4** in DMSO-d6

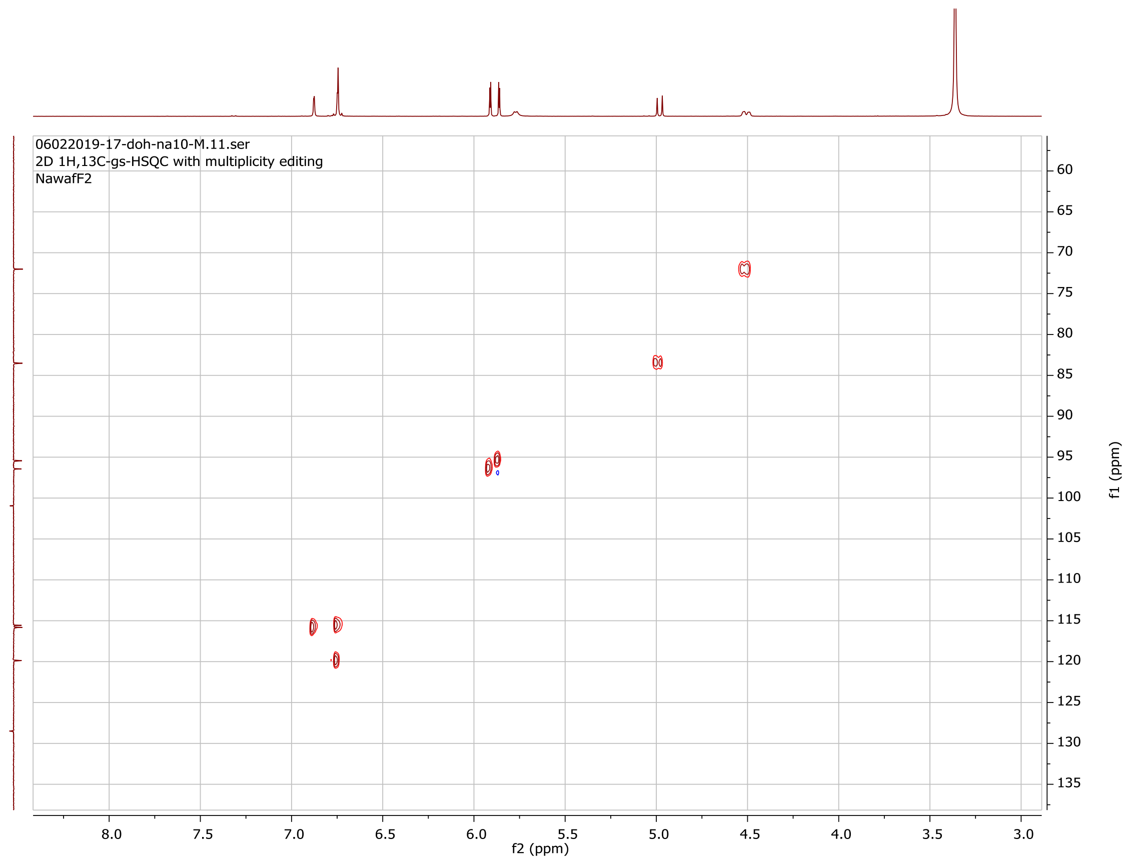

Figure S12. HSQC spectrum of **4** in DMSO-d6

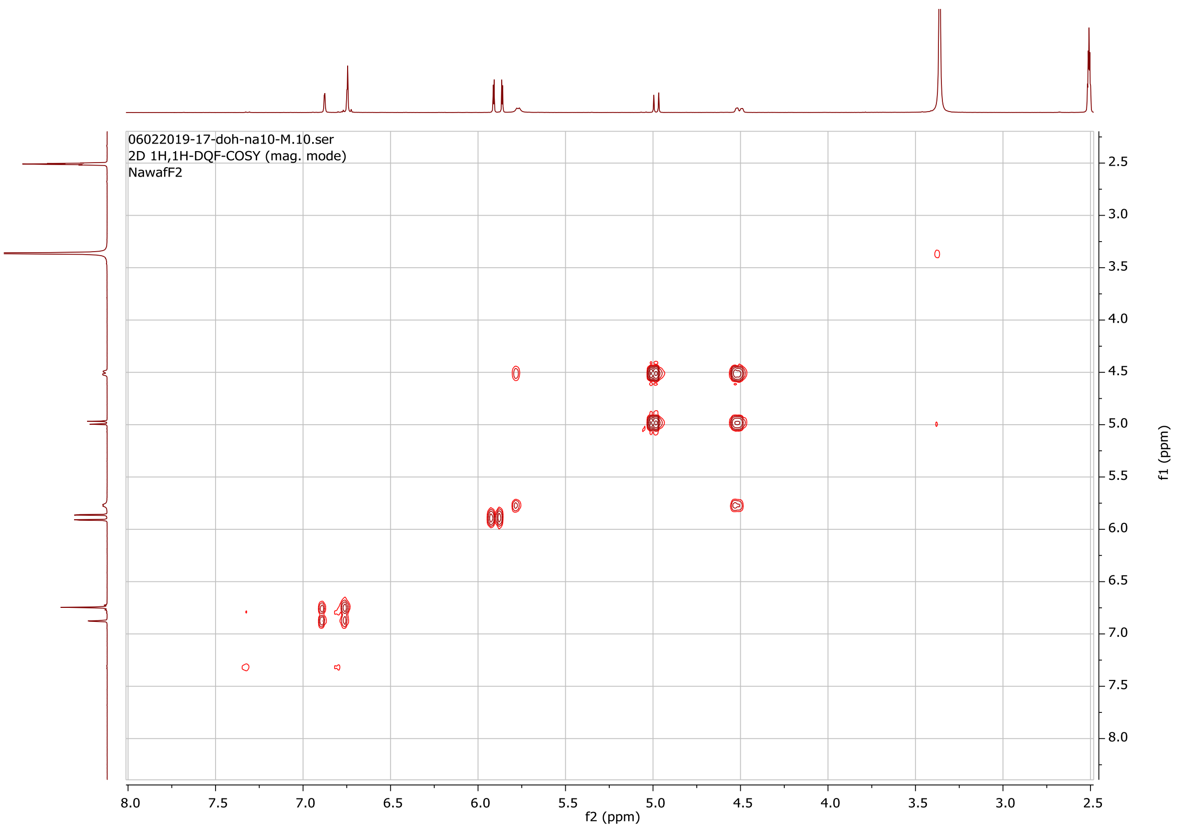

Figure S13. ^{1}H-^{1}H NMR spectrum of **4** in DMSO-d6

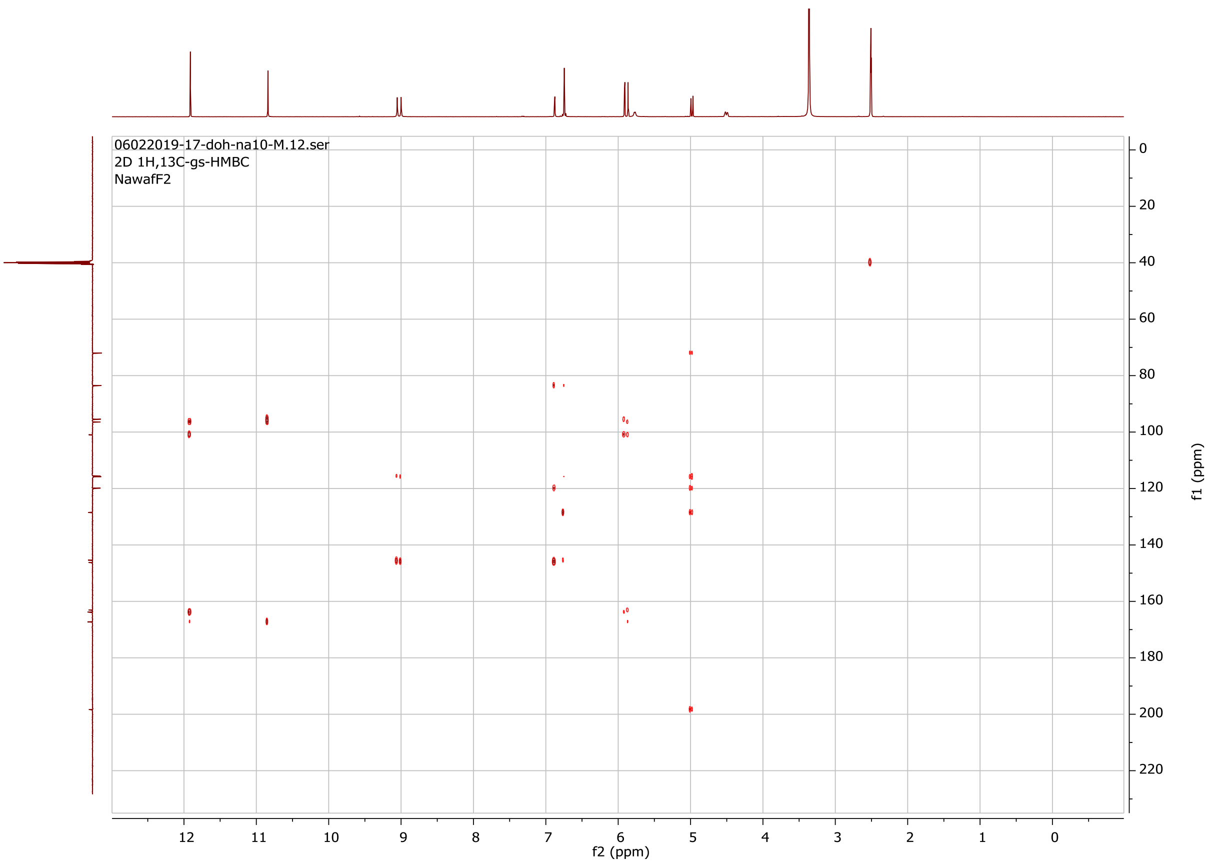

Figure S14. HMBC spectrum of **4** in DMSO-d_6
